# Another Coroner’s Jury Report

**Published:** 1888-10

**Authors:** 


Another Coroner's Jury Report.—We clip the following from the *Appeal* of the 3d ult., as another evidence of imbecility, in matters medical, of the average coroner's jury: "The investigation developed the fact that the dead woman's skull was cracked, exposing the brain. The mother, husband and little child of the dead woman were all examined by the jury, but their evidence failed to show the cause of the strange opening in the skull. There being no further evidence in sight, the jury retired for deliberation, and returned its verdict, which was that the woman died suddenly from a natural cause, produced by an expansion of the skull. Perhaps the knowledge of science displayed by the jury is all right, but, if so, the public, and at least a respectable following in the profession, would like to know it. An *Appeal* reporter stated the facts to one of the foremost physicians and surgeons in the city, and he laughed heartily at the proposition. He further stated that the idea of a skull parting in such a manner, without a violent cause, was preposterous and too absurd for even a moderately intelligent man to credit. If the verdict were found correct, then the medical profession would have a new and highly important field for study open to them."

